# Methylomic signature of lithium response in bipolar disorder

**DOI:** 10.1192/j.eurpsy.2024.468

**Published:** 2024-08-27

**Authors:** C. Bourdon, C. Courtin, F. Bellivier, B. Etain, C. Marie-Claire

**Affiliations:** ^1^Université Paris Cité, Inserm, Optimisation Thérapeutique en Neuropsychopharmacologie; ^2^Hôpitaux Lariboisière-Fernand Widal, GHU APHP.Nord – Université de Paris, Département de Psychiatrie et de Médecine Addictologique, Paris; ^3^Fondation Fondamental, Créteil, France

## Abstract

**Introduction:**

Bipolar disorder (BD) is a chronic and severe psychiatric disorder, characterized by the alternance of episodes of (hypo)-mania and major depression. Lithium (Li) is the first-line treatment for BD but unfortunately response to Li is highly variable: after at least two consecutive years of treatment, only a fraction of patients receiving Li will display significant improvement in the frequency and/or severity of mood recurrences. This interindividual variability of treatment response is difficult to predict, in the bipolar disorder context. This could be determined by genetic factors still misidentified by available genetic studies. In addition, no clinical or biological markers are available to reliably define eligibility criteria for a lithium treatment in bipolar disorder. A consequence is a long process of therapeutic trials (18-24 months) to phenotype Li response, delaying the stabilization.

**Objectives:**

To identify objective biomarkers of the prophylactic response to lithium in order to improve patient care and propose therapeutic alternatives to patients who do not respond to lithium.

**Methods:**

Using a genome-wide methylomic approach, and then logistic regressions incorporating as covariates the different types of treatments, we were able to identify differentially methylated regions (DMRs) whose methylation difference between responders and non-responders was not impacted by co-prescribed treatments. Then, we used Methylation Specific High-Resolution Melting (MS-HRM), a PCR based method than can be implemented in any medical laboratory at low cost and with minimal equipment, to estimate methylation proportion of 9 DMRs in 61 samples of bipolar patients.

**Results:**

In the sample of 61 individuals with BD, the 9 MS-HRM-measured DMRs combined with clinical variables (age, sex, cigarette smoking status, lifetime number of hospitalizations, age at onset of BD, polarity at onset, psychotic symptoms at onset, family history of BD, lifetime alcohol/cannabis misuse, panic disorders, Li prescribed as the first mood stabilizer (vs 2nd or 3rd choice)) correctly classified 83,6% of individuals as good or non-responders (n=61, prophylactic response phenotype defined using the “Alda” scale). Excluding the partial responders, the percentage of correctly classified individuals is as high as 100% (n=43, 18 non responders and 25 responders). The AUC are respectively AUC=0.913 and AUC=1.0.

**Conclusions:**

The MS-HMR method allow to identify the response status of individuals with BD with 9 DMR. These DMRs discriminate good from non-responders and can be used in combination with clinical variables.

**Disclosure of Interest:**

None Declared

